# Comparison of Different Anthropometric Indicators for Assessment of Nutritional Status Among Adolescent Girls in an Urban Resettlement Colony in New Delhi: A Cross-Sectional Study

**DOI:** 10.7759/cureus.37242

**Published:** 2023-04-07

**Authors:** Archismita Santra, Sanjay Rai, Puneet Misra, Kapil Yadav, Kiran Goswami, Gurdeep Kaur

**Affiliations:** 1 Centre for Community Medicine, All India Institute of Medical Sciences, New Delhi, New Delhi, IND; 2 Department of Dietetics, All India Institute of Medical Sciences, New Delhi, New Delhi, IND

**Keywords:** nutritional status, stunting, mid upper arm circumference(muac), body mass index(bmi), bmi-for-age, malnutrition, anthropometric indicators, overnutrition, undernutrition, adolscent girls

## Abstract

Background

In India, 44.8% of adolescent girls are under-nourished, while about 8%-13% of girls are overweight. Though several studies have been done regarding the nutritional status of adolescent girls over the years, there have been no significant changes. Also, there are several different anthropometric indicators for nutritional status assessment, due to which there are huge variations in the prevalence of malnutrition across different studies. So the objectives of this study were to determine the prevalence of malnutrition using different anthropometric indicators and compare them.

Methods

A random sample of 426 girls was taken from Health Management Information Systems (HMIS) of the Centre for Community Medicine (CCM), All India Institute of Medical Sciences (AIIMS), and a semi-structured questionnaire was administered among 386 of them to determine associated factors. Height, weight, and mid-upper arm circumference (MUAC) were measured for 386 girls, and BMI for age and height for age z scores were calculated using WHO AnthroPlus. Mid-upper arm circumference for age z scores (MUAC for age) were calculated using Lambda-Mu-Sigma (LMS) charts by the CDC for girls aged one year to 20 years.

Results

It was found in this study that using BMI for age z scores (BAZ), 33.4% of the adolescent girls were malnourished; 18.9% (95% CI 15.1-23.2) being underweight, 10.6% (95% CI: 7.7-14.1) being overweight, and 3.9% (95% CI: 2.2-6.3) were obese. While using BMI solely as an indicator, the prevalence of thinness was 51.8% (95% CI: 46.9-56.9), while that of overweight and obesity was 10.6% (95% CI: 5.7-11.5) and 1.0% (95% CI: 0.2-2.6), respectively. The prevalence of under-nutrition by MUAC for age z scores was 53.4% (95% CI: 48.2-58.4), and that of over-nutrition was 1.8% (95% CI: 1.0-3.7). BMI for age z scores positively and strongly correlated with both MUAC and MUAC for age z scores and had a significant association with both on univariable linear regression. Though there was a negative correlation between BMI for age z scores and height-for-age z scores, it was not significant. Height-for-age z scores, even though positively correlated with MUAC for age z scores, the correlation was not that strong.

## Introduction

Adolescents consist of about 21.2% of India’s population [1]. During adolescence, the rate of physical growth is second only to the first year of life and one of the most important periods of growth spurts where targeted interventions can be given to improve the overall health of the individuals [2]. Malnutrition still remains a serious health problem among adolescents in India, especially among girls, despite the implementation of several programs and schemes like Rashtriya Kishor Swasthya Karyakram (RKSK), Rajiv Gandhi Scheme for Empowerment of Adolescent Girls (RGSEAG Sabla), Integrated Child Development Services (ICDS), Prime Minister’s Overarching Scheme for Holistic Nourishment (POSHAN) Abhiyaan with importance to adolescent health [1,3-5]. It has been found that the prevalence of thinness among adolescent girls in urban India ranged from 15% to 74%, while that of being overweight was about 8% to 13%, primarily due to the varied standards of assessment of nutritional status across different studies [6-20]. National Family Health Survey-4 (NFHS-4) states that in the age group of 15-19 years, 41.9% of girls were undernourished, and 4.2% of girls were overweight or obese [21]. Following the life cycle approach, under-nourished women give birth to low birth weight babies who, in turn, may have under-nourished childhood and adolescence. Such females might have poor pregnancy outcomes when they become pregnant [22,23]. Even overweight and obese adolescents may suffer from non-communicable diseases like hypertension and diabetes in adulthood [23]. Various anthropometric indicators have been routinely used for the assessment of nutritional status among adolescents, which include height, weight, BMI, mid-upper arm circumference (MUAC), BMI for age z scores, weight for age z scores, height for age z scores and the results vary across the studies according to the standards used for assessment. Also, there are several standards by the WHO, CDC, Indian Academy of Paediatrics (IAP), and Indian Council of Medical Research (ICMR) for assessment of nutritional status of adolescents [24-30]. While WHO, CDC, and IAP use z scores of anthropometric indicators as standards for the assessment of the nutritional status of children and adolescents, ICMR uses BMI-based criteria, i.e., thinness (BMI <18.5 Kg/m2) and overweight (≥25.0 Kg/m2) or obesity (≥29.9 Kg/m2) for assessment of nutritional status. This BMI-based criteria has also been used in NFHS for the age group of 15-19 years [21]. The WHO BMI-for-age z-score charts for 5-19 years were constructed in 2007, which is comparatively more recent than IAP z-score charts for 5-18-year-old girls for which affluent data collected from children in 1989 was used [27]. CDC BMI for age z scores has been found to be the best predictor of metabolic syndrome, which can be considered a proxy of over-nutrition when both boys and girls are considered together [31]. However, WHO BMI for age z scores are considered to be better predictors of metabolic syndrome or over-nutrition solely for girls [31]. 

When BMI estimates of NFHS-3 and NFHS-4 were revised using BMI for age z scores (WHO 2007 growth reference), it was found that thinness among 15-19-year-old girls was 9.9% and 9%, respectively, compared to 46.8% and 41.9%, respectively, with BMI-based criteria. There was wide variation among the states with pockets of a double burden of malnutrition [32]. Thus, it shows that BMI-based criteria overestimate the prevalence of thinness than using BMI for age z scores. Also, in the Comprehensive National Nutrition Survey 2016-2018 (CNNS 2016-2018), where BMI for age z scores was used for the assessment of the nutritional status of adolescents, the prevalence of thinness among adolescent girls by BMI for age was 18.9% which is much low compared to NFHS [33]. There have been very few studies in India where MUAC was used as an anthropometric indicator for determining the nutritional status of adolescent girls. However, apart from CNNS, we did not find studies from India using MUAC for age z scores as a measure for the assessment of nutritional status. Though MUAC is easy to measure in community-based studies, calculating z scores involves complex calculations using Lambda-Mu-Sigma (LMS) charts for MUAC for age z scores. Also, cut-offs of MUAC for determining under-nutrition or over-nutrition among adolescent girls vary widely across age groups and populations and need to be validated [34, 35]. Therefore, standardization for age and sex can be done by using BMI for age z scores, MUAC for age z scores, and height for age z scores rather than solely BMI, MUAC, and height as standards to assess nutritional status. Thus, this study aimed to assess the prevalence of malnutrition among adolescent girls in an urban resettlement colony in Delhi using different anthropometric indicators and compare them.

## Materials and methods

This cross-sectional study was conducted among adolescent girls aged 10-19 years in an urban resettlement colony at Dakshinpuri Extension in Delhi, the urban field practice area (UFPA) under the CCM, All India Institute of Medical Sciences (AIIMS), New Delhi, from May to August 2019. AIIMS UFPA consists of 10 blocks at Dakshinpuri Extension with a population of 36,868 people, according to HMIS for UFPA maintained at CCM, AIIMS. HMIS maintains all essential health-related information of the habitual residents of UFPA. Two blocks with populations of 3545 and 3446 were conveniently selected for this study out of ten blocks. The estimated prevalence of under-nutrition was taken to be 50% due to massive variability in the prevalence estimates of under-nutrition among adolescent girls, and absolute precision was assumed to be 5%. The calculated sample size for the estimation of under-nutrition was 426, assuming a 10% non-response rate. Similarly, the prevalence of over-nutrition was taken to be 9% with 5% absolute precision for sample size calculation. Taking into account 10% non-response response rate, the sample size for the assessment of over-nutrition was calculated to be 140. Thus, the minimum required sample size was taken to be 426 for the assessment of nutritional status of adolescent girls. A sampling frame of 557 adolescent girls was obtained from the two selected blocks from HMIS for UFPA. Simple random sampling without replacement was done to obtain the sample size of 426 adolescent girls. All adolescent girls, irrespective of marital status, were included in the survey. The girls who could not comprehend the interviewer's instructions or could not be contacted despite three successive visits to their houses on consecutive days were excluded from the study. Every household mentioned in the sample was visited. A semi-structured questionnaire was administered to all the study participants. The participants' anthropometric indicators were measured, including height, weight, and MUAC. Weight was measured twice on the same visit, and the mean weight was taken. MUAC was measured up to the nearest 0.1 cm on bare skin using inextensible tape in a relaxed position of the non-dominant arm at the mid-point of the line joining acromion and olecranon process [26].

The BMI of all the participants was calculated and classified according to WHO BMI criteria. Those with BMI less than 18.5 kg/m2 were classified as thin, those with a BMI of 25 kg/m2 to 29.9 kg/m2 were classified as overweight, and those with BMI ≥ 30 kg/m2 were classified as obese. WHO standards of BMI for age z scores and height for age z scores for 5 to 19-year-old adolescent girls were also used to assess nutritional status and were calculated using WHO AnthroPlus software. Those who had BMI for age z scores <-2 SD were classified as thin, and those who had BMI for age z scores ≥+1 SD to ≤+2 SD were classified as overweight, and scores >+2 SD were considered obese [26, 29]. As per WHO, stunting was defined as height for age z scores less than -2 SD from the median. MUAC for age z score ranging from -2 SD to +2 SD was considered normal. Reference LMS charts for MUAC for one-year to 20-year-old girls, which were derived based on the population used in CDC 2000 BMI for age growth charts [30], were used to calculate MUAC for age z scores using the following formula by coding for age separately, as standardized LMS chart for Indian population is not available till now. MUACz=[(MUAC/M)L-1]/(L*S), where MUACz = MUAC for age z score; L= power transformation to eliminate skewness; M=median; and S=Coefficient of variation.

Ethical considerations

Ethical approval for the study was obtained from Institute Ethics Committee, All India Institute of Medical Sciences, New Delhi (IECPG-66/28.02.2019, RT-40/27.03.2019). Assent was obtained from adolescent girls aged below 18 years, along with written informed consent from their legally authorized representatives. For adolescent girls over 18 years, written informed consent was taken from the study participants. 

Data management and analysis

The semi-structured questionnaire was pretested before starting the study. The height and weight of each participant were entered in WHO AnthroPlus software for getting BMI for age and height for age z scores. LMS charts for MUAC given by CDC for ages one year to 20 years were used to calculate MUAC for age z scores in MS Excel. Data from the WHO AnthroPlus and MUAC for age z scores for all the participants were merged after checking for any missing data and analyzed finally using Stata 12.1. For descriptive statistics, percentage and frequency were used for categorical variables, and mean and SD were quoted for quantitative variables. For the association between independent and dependent quantitative variables, linear regression was used. Also, for quantitative variables, scatter plots were used to see a correlation. A p-value less than 0.05 was considered to be significant.

## Results

**Figure 1:**
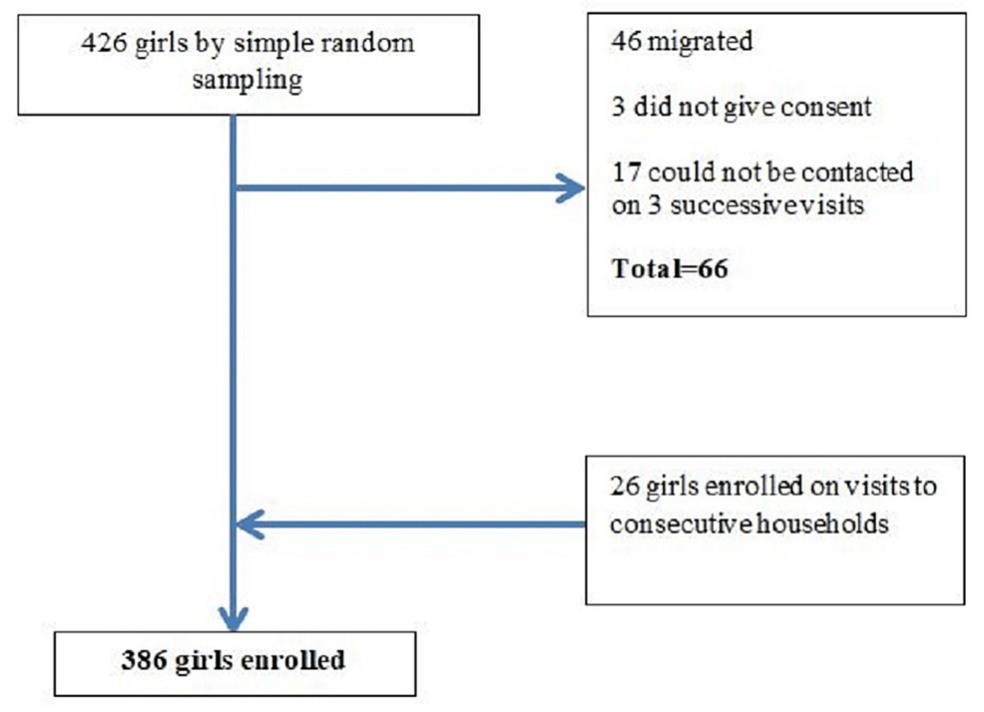
Flowchart of study participants.

A random sample of 426 adolescent girls was retrieved from HMIS, out of whom 386 girls participated in the study, with a non-response rate of 9.4%. When an adolescent girl from the obtained sample was not available for three successive visits on consecutive days or had migrated, the next household in the same building was visited. If there were more than one adolescent girl in that household, only one girl was selected for the study by lottery method to meet the adequate sample size. In this process, 66 girls were excluded from the study, and 26 more girls were included from consecutive households (Figure 1).

The mean age of the study participants was 14.8 years (SD 0.13). The majority (58.5%) of the girls belonged to late adolescence (15-19 years) and had attained menarche (15-19 years). A total of 98.7% of the girls were currently going to school, and none of them were married. Most of them were Hindu (98.9%) and belonged to extended families (67.6%). The parents of the majority of the girls were educated up to middle school. The families of most of the girls were headed by their fathers (86.8%), and most of their fathers were engaged in semi-skilled work (56.7%). The majority of the girls belonged to upper-lower socioeconomic status (59.3%) according to the Modified Kuppuswamy Scale (2018) (Table 1). 

**Table 1:** Descriptive characteristics of study participants. *Fathers of 12 girls were dead and fathers of six girls were unemployed. Mothers of two girls were dead. ^#^Socio-economic status was assessed by Modified Kuppuswamy Scale (2018).

Age category	Frequency (N=386)	Percentage (%)
Early adolescence (10-14 years )	160	41.5
Late adolescence (15-19 years)	226	58.5
Attained Menarche	N=386	%
Yes	283	73.1
No	103	26.9
Schooling status	N=386	%
Currently going to school	381	98.7
Currently not going to school	5	1.3
Religion	N=386	%
Hindu	382	98.9
Muslim	4	1.1
Type of family	N=386	%
Nuclear	125	32.4
Extended	261	67.6
Number of siblings	N=386	%
Up to one	126	32.6
More than one	260	67.4
Birth order	N=386	%
One	153	39.6
Two	143	37.1
More than two	90	23.3
Head of family	N=386	%
Father	335	86.8
Mother	13	3.4
Others	38	9.8
Father’s education*	N=374*	%
Up to middle school	291	77.8
Above middle school	83	22.2
Mother’s education	N=384*	%
Up to middle school	288	75.3
Above middle school	95	24.7
Father’s occupation	N=368*	%
Skilled	17	4.6
Semi-skilled	209	56.7
Unskilled	142	38.7
Mother’s occupation	N=384*	%
Skilled	8	2.1
Semi-skilled	46	12.0
Unskilled	330	85.9
Parent’s marital status	N=386	%
Married	363	94.0
Others	23	6.0
Socio-economic status^#^	N=386	%
Upper middle (2)	27	7.0
Lower middle (3)	130	33.7
Upper lower (4)	229	59.3

In this study, when BMI was used as an indicator for assessment of malnutrition, 51.8% of girls were thin (BMI < 18.5 Kg/m2), 8.3% of girls were overweight (BMI: 25.0-29.9 kg/m2), and 1% were obese (BMI ≥30.0 kg/m2 ) (Table 2). However, according to BMI for age z score as an anthropometric indicator, it was found that 18.9% of girls were thin, 10.6% were overweight, and 3.6% of girls were obese (Table 3). When classified by MUAC for age z scores, 53.4% of girls were undernourished, while only 1.8% were overnourished. It was observed that 24.6% of the girls were stunted using height for age z scores (Table 3).

**Table 2:** Prevalence of malnutrition among study participants using WHO classification for BMI.

BMI (Kg/m^2^)	Frequency (N=386)	Percentage (%)	95% CI
Normal (18.5 to 24.9 )	150	38.9	33.9-43.9
Thinness (< 18.5)	200	51.8	46.9-56.9
Overweight (25.0 to 29.9)	32	8.3	5.7-11.5
Obese (≥30.0)	4	1.0	0.2-2.6

**Table 3:** Prevalence of malnutrition and stunting among study participants using age-standardized z scores for different anthropometric indicators. MUAC: Mid-upper arm circumference.

BMI for age z scores	Frequency (N=386)	Percentage (%)	95% CI
Normal	257	66.6	61.6-71.2
Thinness	73	18.9	15.1-23.2
Overweight	41	10.6	7.7-14.1
Obese	15	3.9	2.2-6.3
MUAC for age z scores			
Over-nourished	7	1.8	1.0-3.7
Normal	173	44.8	39.7-49.9
Under-nourished	206	53.4	48.2-58.4
Height for age z scores			
Normal	291	75.4	70.8-79.6
Stunted	95	24.6	20.4-29.2

**Figure 2:**
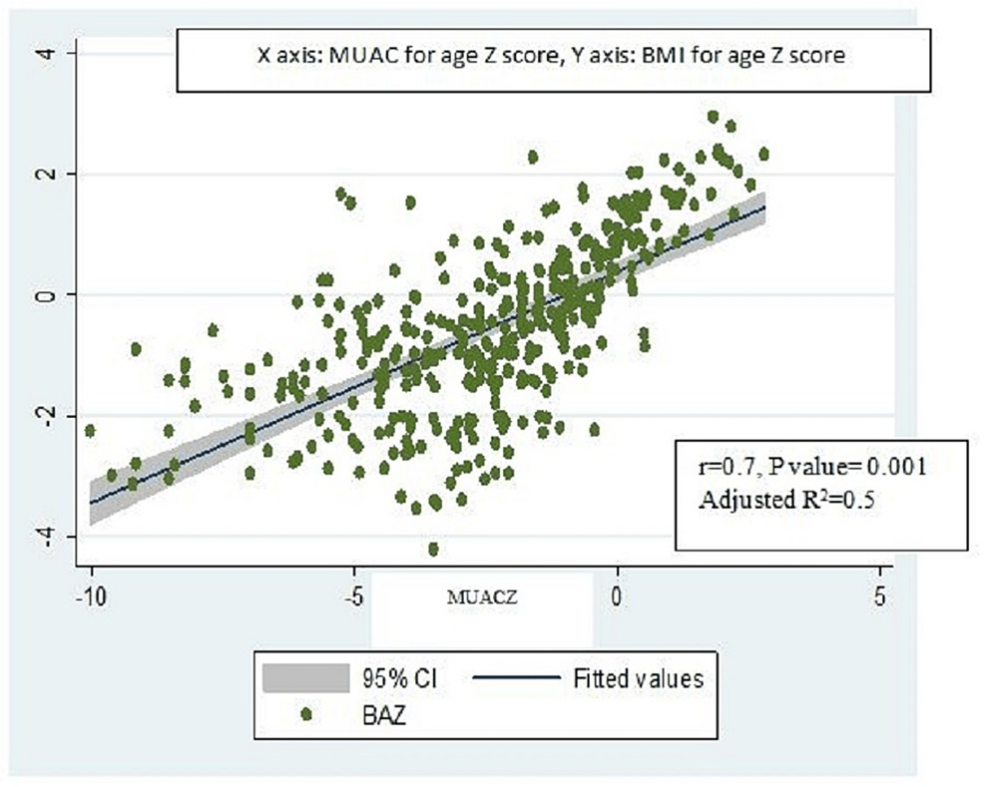
Scatter plot for correlation between MUAC for age z scores and BMI for age z score. MUACZ: Mid-upper arm circumference for age z scores; BAZ: BMI for age z score.

**Figure 3:**
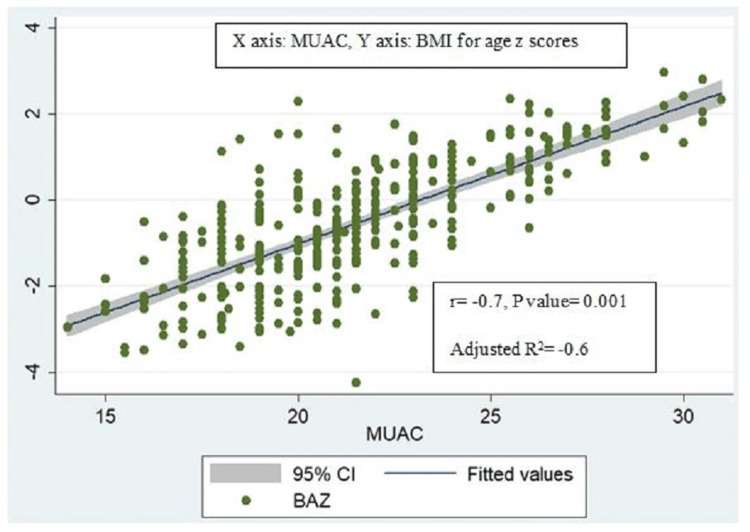
Scatter plot for correlation between BMI for age z scores and MUAC. MUAC: Mid-upper arm circumference; BAZ: BMI for age z score.

**Figure 4:**
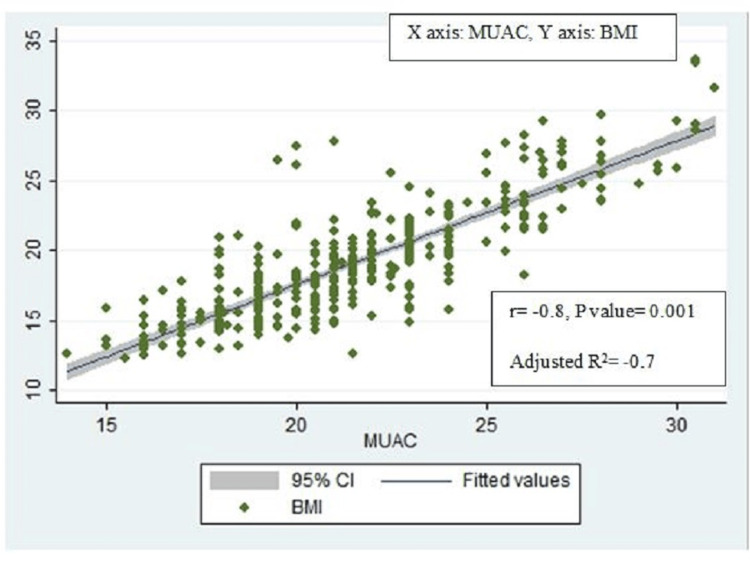
Scatter plot for correlation between BMI and MUAC. MUAC: Mid-upper arm circumference.

**Figure 5:**
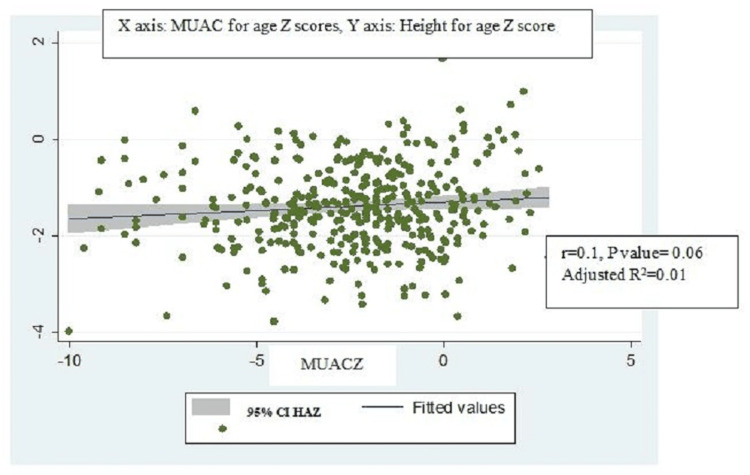
Scatter plot for correlation between MUAC for age z scores and height for age z score. MUACZ: Mid-upper arm circumference for age z scores; HAZ: Height for age z score.

**Figure 6:**
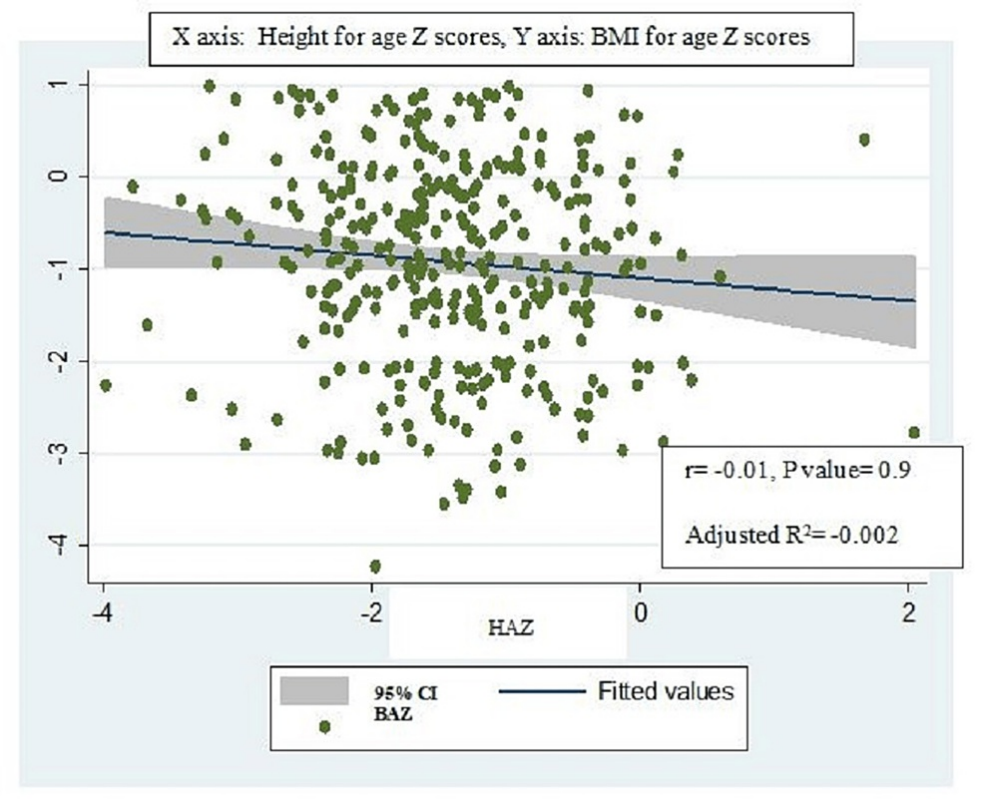
Scatter plot for correlation between BMI for age z scores and height for age z score. BAZ: BMI for age z score; HAZ: Height for age z score.

BMI-for-age z scores were positively and strongly correlated with MUAC-for-age z scores (r=0.7, p-value=0.001, adjusted R2=0.5) (Figure 2) and significantly associated with each other (ꞵ coefficient = 0.6, 95% CI: 0.5-0.7). BMI for age z scores also similarly had a strong positive correlation with MUAC (r=0.7, p-value=0.007, adjusted R2= 0.6) (Figure 3) and was also significantly associated with MUAC (ꞵ coefficient = 0.3, 95% CI: 0.2-0.4) on univariable linear regression (Table 4). However, though BMI was strongly correlated with MUAC (r=0.8, p-value=0.001, adjusted R2=0.7) (Figure 4), they were not significantly associated with each other (ꞵ coefficient=1.0, 95% CI: 0.9-1.1). Height-for-age z scores, although positively correlated with MUAC-for-age z scores, were not strong (r= 0.1, p-value=0.06, adjusted R2=0.01) (Figure 5). There was a negative correlation between BMI for age z scores and height for age z scores, but it was not significant (r=-0.01, p-value=0.9, adjusted R2=-0.002) (Figure 6). 

**Table 4:** Univariable linear regression for the association of BMI for age z scores with other anthropometric indicators MUAC: Mid-upper arm circumference.

Anthropometric indicators	ꞵ coefficient	95% CI	P-value
MUAC	0.3	0.2-0.4	0.001
MUAC for age z score	0.6	0.5-0.7	0.001
Height for age z score	-0.01	-0.2-0.1	0.8

## Discussion

The prevalence of malnutrition among adolescent girls using the WHO BMI criteria was found to be 62.1%, of whom 58.1% were thin, 8.3% were overweight, and 1% were obese. Wherein by using the WHO BMI for age z scores, the total prevalence of malnutrition was found to be 33.4%, where the prevalence of thinness was 18.9% and that of overweight and obesity was 10.6% and 3.9%, respectively. In this case, the WHO BMI-based criteria overestimate the prevalence of undernutrition compared to WHO BMI for age z scores. A study by Bhargava M et al. had similar findings when estimates of NFHS-4 using BMI-based criteria were converted to BMI for age z scores [32]. In our study, the prevalence of overweight and obesity (14.5%) is slightly higher. In comparison, undernutrition (18.9%) is grossly lower than the estimated values used to calculate the sample size for the study from the literature review [6-20]. The prevalence of thinness was also found to be much less than NFHS-4 (2015-2016). One probable reason was the use of BMI alone as the measure for nutritional status assessment in NFHS-4 [21]. CNNS (2016-2018), which had a similar methodology of considering the WHO BMI for age z scores as the measure for the assessment of the nutritional status of adolescent girls like our study, had a similar prevalence of undernutrition (18.9%) [31]. However, the prevalence of overweight/obesity in the present study is higher than in NFHS-4 and CNNS. This might be area-specific and depending on the socio-economic and socio-demographic factors in that area, as this study has been conducted in an urban area of Delhi. At the same time, both nationwide surveys have larger representative samples from different parts of the country. In a study by Acharya A et al. among girls of 10-19 years of age in an urban slum of Delhi in 2005, 74% of the girls were reported to be underweight using the WHO BMI-based criteria [6]. In comparison to our study, which reports the prevalence of thinness to be 58.1% using the same criteria as above, this fall in the prevalence of thinness in the last 15 years, which can probably be explained by improvement in the educational and socio-economic status of the people over the years. In a study conducted by Pramanick P et al. in 2013-2014 in Hooghly among 11-18-year-old girls, 15% of girls were observed to be thin (by the WHO BMI for age criteria), which is similar to this study using similar methods [14].

According to CNNS, among girls aged 10-19 years, 23.2% had MUAC below -2 SD [34]. In the present study, this was found to be 53.4%. This might be explained by a smaller sample size in our study compared to that of CNNS. A positive correlation was found between MUAC for age z scores and BMI for age z scores (r=0.7) and between MUAC and BMI for age z scores (r=0.7), and BMI for age z scores were significantly associated with both MUAC for age z scores as well as MUAC. There was also a strong positive correlation between BMI and MUAC (r=0.8), but they were not significantly associated with each other. In a study conducted among adolescent girls in Pakistan conducted by Asif M et al. in 2016 in Islamabad and Rawalpindi among 5-14 years children [34], a similar correlation between MUAC and BMI was found for girls (r=0.71, p value=0.01). A study conducted among 10-19-year-old boys in Chetla, West Bengal, by Dasgupta A et al., found a strong correlation between BMI and MUAC (r=0.82, p value=0.00) [35]. Even for using MUAC as a proxy indicator for MUAC for age z scores, the MUAC cut-offs for determining nutritional status among adolescent girls should be validated across age groups, gender, and in the Indian population, and MUAC cut-offs for assessing the nutritional status of the adolescent population is still not available specifically for India [36,37]. Though MUAC-for-age z score is also a good measure of malnutrition among adolescents as it can be measured in persons whose height and weight cannot be measured, due to mathematical corrections which have to be applied, using MUAC for age z scores becomes cumbersome in community-based studies. However, MUAC takes into account the body fat mass, which BMI does not consider. Also, LMS values for calculating MUAC for age z scores for the Indian population need to be standardized for using MUAC for age z scores in more extensive surveys. However, it is seen in this study that BMI for age z scores correlate similarly and strongly with both MUAC and MUAC for age z scores. CNNS and NFHS were found to be grossly different in methodologies and units of measurement for the assessment of the nutritional status of adolescents. Thus, the units and measurement methods need to be standardized to carry out more large-scale surveys among adolescents in India to assess their nutritional status, which can be compared over the years. The strengths of this study are that different anthropometric indicators for the assessment of malnutrition were studied together and correlated. However, triceps skin fold thickness was not studied here because there is no standard cut-off available for the classification of the nutritional status of adolescents according to triceps skin fold thickness which is a limitation of this study. This study strictly used two blocks of an urban resettlement colony in Delhi and thus cannot be generalized to the entire population, which is another limitation of this study.

## Conclusions

Our study shows that the WHO BMI-based criteria overestimate the prevalence of under-nutrition among adolescent girls compared to the WHO BMI-for-age z scores. Also, over-nutrition among adolescent girls was higher than expected, and the prevalence of thinness was lower than expected in an urban area in Delhi. Both MUAC and MUAC-for-age strongly correlate with BMI-for-age z scores. Thus MUAC cut-offs, if validated in the Indian population for determining malnutrition, can be used as a proxy indicator for MUAC-for-age z scores in large studies for the ease of mathematical calculation. Data from national surveys and other studies can be compared only if the age groups and measurement methods are standardized. Thus, standardized anthropometric indicators with respect to age and sex should be used for the assessment of nutritional status among adolescent girls for large-scale surveys.
